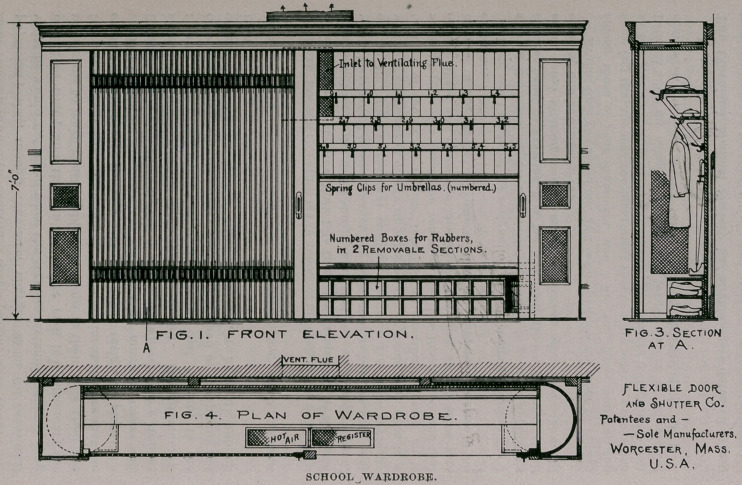# Topics of the Month

**Published:** 1895-04

**Authors:** 


### TOPICS OF THE MONTH.

The Buffalo General Hospital has received the munificent gift of $55,000, which is to be applied in the building of a new structure. This royal donation was made by four of Buffalo's most philanthropic women. Mrs. George B. Gates contributed $40,000 of the amount, and her three daughters—namely, Mrs. William Hamlin, Mrs. Charles W. Pardee and Miss Elizabeth Gates, each added $5,000 to the sum.

There is much cause for congratulation in this splendid liberality of these sterling women. In the first place Buffalo has great need of improved hospital accommodations. At present there is no adequate structure for the reception of patients that may be properly classed as general in character. Buffalo is rapidly increasing in population, and its hospitals should keep pace with the progress of events.

It is understood that a new general hospital has been contemplated for some time. The gift of Mrs. Gates and her daughters will make a substantial beginning in the erection of an appropriate building. Buffalo deserves a charity of this kind that ought to cost $1,000,000. Who of Buffalo's philanthropic citizens will supplement the Gates' fund, increasing it to the amount named? The princely gift of these charitable women ought to bring the blush of shame to some of our wealthy citizens, and stimulate them to prompt action in swelling the fund to an amount that would put in immediate process of construction a building such as the picture we present, indicates.

It is stated that action will be taken at once looking to the erection of one wing of the new structure in accordance with plans drawn by Mr. George Cary, architect, of this city. The plan contemplates the erection of a quadrangular group of buildings as exhibited in the illustration, which shows the High-street elevation.

Mrs. Gates, principal donor of the fund mentioned, is now eighty years old. She takes an active interest in the prosperity of Buffalo, and especially in charitable work, and we hope that she and her three daughters may be spared many years to witness and enjoy the fruition of their princely charitable enterprise.

---

Though our contemporaries, in a number of instances, refer to the "New York State Medical Society," we beg to inform them that

THE BUFFALO GENERAL HOSPITAL.

From THE ILLUSTRATED BUFFALO EXPRESS. Copyrighted by Geo. E. Matthews & Co.

there is no such organization. There is a New York State Medical Association, which was organized a few years ago and has its headquarters in the city of New York; but the "Medical Society of the State of New York" is the official designation of a body organized in 1806 and that holds its annual meetings at Albany. Heretofore it has met on the first Tuesday of February, but at its last meeting it decided to hold its next one on the last Tuesday of January, 1896.

---

A BILL has been introduced into the legislature, says the *Medical Record*, that threatens the Craig Colony for Epileptics with disruption. Mr. Otto Kelsey, the member from Livingston county, in which the colony is located, is the reputed author of the bill. It is proposed to remove the present managers and appoint twelve others in their places selected on a different plan. The Rochester newspapers are opposing the bill with considerable vehemence and we think with much propriety. The committee of legislation of the Medical Society of the State of New York could here find a field for good work.

---

IN THESE days, when the trend of medical opinion is everywhere in favor of improved methods of medical education; and especially when it favors advances in preliminary education, lengthened terms in medical colleges and separate examination by the state, it would be difficult to persuade us that any medical journal would be found in opposition to these principles. Listen, however, to the following echoes from the *North American Medical Review* (Kansas City), February, 1895:

> All physicians interested in teaching medicine and elevating the standard of medical education through the colleges, *should discourage* the further establishment of medical examining boards. These boards are not constituted from the brainy element of the profession, but from medical politicians with a pull; and to transfer the licensing power to them is to take away a time-honored prerogative from the colleges and transfer it to politicians, or placing politics versus science.
>
> The medical practice act of the State of Tennessee has been declared unconstitutional by Judge Anderson of the Supreme Court of that State, *as it tended to prohibit physicians from advertising.* Under this decision the medical practice act of Missouri and other states could be declared null and void.

These paragraphs would seem more appropriate if written a century ago, but even then they would have been very much out of place

A HITHERTO unknown constituent of atmospheric air has been discovered, to which the name "argon" has been given. Two members of the Royal Society of London, Dr. Ramsay and Lord Rayleigh, are credited with this remarkable and we hope useful discovery. Chemists have long ago told us that hydrogen obtained from chemical compounds differs from hydrogen obtained from the air, the former being lighter than the latter. This difference is not due to impurities, but to the presence in the air of some substance greater in density than hydrogen. The density of argon is stated to be about 20; that it is two and a half times more soluble in water than is nitrogen; that its special characteristic is inertness, and that all attempts to induce its chemical combination with other bodies thus far have failed. An opportunity seems to be presented for original investigation in regard to this somewhat mysterious substance, but one that promises great usefulness.

---

DURING the past week, says the *Boston Medical and Surgical Journal* of March 21, 1895, a students' examination was held by the regents of the university at Albany for such first-year students of medicine as had not been graduated from a registered college, or satisfactorly completed not less than a three years' course in a registered academy or high school. The studies comprised in the examination are: arithmetic, elementary English, geography, spelling, United States history, English composition and physics. During the examination referred to, it was discovered that two of the applicants had appeared under assumed names; one representing a student of Bellevue Hospital Medical College and the other a student of the College of Physicians and Surgeons. It is said that those concerned in the fraud will be black-listed in all the medical colleges of the state, and that perhaps they may be prosecuted criminally by the Medical Society of the County of New York. The wisdom of placing the control of medical education and practice in the hands of the regents thus again is demonstrated.

---

THE JOURNAL has observed a tendency on the part of a number of its contemporaries to borrow its paragraphs in whole or in part, in idea or substance, without due credit. This is a journalistic courtesy which we always accord and which we claim in return as just and reciprocal. We are only too glad to find that our efforts are appreciated and our work appropriated, but we would like to have a recognition of the same, and, most of all, a maintenance of the ordinary amenities of good journalism.

---

The Journal always takes pleasure in recording inventions that tend to improve schoolhouse sanitation. A novel application of flexible doors has been devised by an inventor in Worcester, Mass., to economize floor areas in the construction of school and other public buildings which, at the same time, provides sanitation and security to clothing at a minimum of cost. The cut on the opposite page shows a school wardrobe, thirteen feet wide and two feet deep, placed against the wall in the hallway, that will provide clothing accommodation for forty or fifty pupils. The hooks, umbrella clips and pigeon-holes for overshoes are in plain view, arranged and numbered vertically in sets to accommodate pupils of all ages.

The flexible doors are constructed of wood moldings, connected by a series of concealed interlocking steel hinges running through their entire width, making them strong and durable. They are provided with good locks that afford security to clothing and valuables. Ventilation is secured in the side and end panels, which are provided with strong wire netting. At the top is an opening of sufficient area to connect with the nearest ventilating flue, thus providing for forced draft, which is desirable. Hot air from furnace or steam pipes may be introduced through a register to dry and warm the clothing during wet or cold weather, which every sanitarian will recognize as a desirable feature.

The doors are constructed so as to run smoothly, easily and without friction. These wardrobes have already been adopted in Brooklyn and other cities. Their cost is from $75.00 upward, according to size. Their comparative low price commends them to school authorities when, in the construction of buildings, the saving of floor areas is an item of importace materially decreasing the cost of construction. But, most of all, they are to be commended on account of their sanitary features.

---

A just rebuke has been dealt the management of the New York Life Insurance Company by Dr. De Lancey Rochester, of Buffalo, the details of which appear in a letter published by Dr. Rochester in the *New York Medical Journal*, January 26, 1895.

The company referred to has sent out a circular to its medical examiners reducing their fee for examination from $5.00 to $3.00.

FIG. 1. FRONT ELEVATION.

FIG. 3. SECTION AT A.

FIG. 4. PLAN OF WARDROBE.

FLEXIBLE DOOR

AND SHUTTER Co.

Patentees and —

— Sole Manufacturers.

WORCESTER, MASS.

U.S.A.

SCHOOL WARDROBE.

The circular states that the company feels that it stands in the same relation toward its examiners that a patient does toward his physician; that, though the latter charges $5.00 for occasional visits he would not do so when the services extended over a long period of time; and that, in the latter case, it is customary for physicians to make considerable reductions in their bills from the fee for single visits. Continuing, the circular intimates that the examiner may accept the new arrangement or resign his place.

Dr. Rochester discusses the subject with much clearness and illustrates his argument by citing examples wherein the examiner saves insurance companies large sums of money. His communication to the *Journal* concludes with a letter that he sent to the company in reply to its impertinent circular, and as it explains not only his position but that of other examiners, we herewith publish it in full:

*New York Life Insurance Company, A. Huntington, M. D., Medical Director:*

DEAR DOCTOR: Permit me to announce my resignation as a medical examiner for the New York Life Insurance Company. At the same time permit me to say that I should think that you, as a medical man, would be ashamed to countenance any such letter as has just been sent out by Dr. Rogers, assistant medical director. You know perfectly well that no physician of any standing whatever would give, even to a member of one of his regular families, an examination such as is required by a life insurance company, for less than $10.00, and that examinations are made for insurance companies at the $5.00 rate because of the amount of business they are supposed to send to the examiner.

Long ago I tendered my resignation to your company because, as I said at the time, it was not worth my while to go to an applicant's office or house to examine him for less than $10.00, but that if the agent cared to bring one to my office *during office hours*, I would examine him at the $5.00 rate.

At that time you asked me, as a favor, to allow my name to remain among those of your examiners, and I did so. Now, however, the mere fact that the medical department should allow such a proposition as has just been made to receive its sanction renders it impossible that I should allow my name to remain any longer as one of its medical examiners. DE LANCEY ROCHESTER, M. D.

---

THE Cleveland Medical Society has unanimously defeated a proposition to make its constitution and by-laws conform to the code of ethics as interpreted by the San Francisco meeting of the American Medical Association. The *Western Reserve Medical Journal*, distinguished for its high journalistic tone, makes some very pointed and able comments on this action of the Cleveland Medical Society. We wish we had space to print the entire editorial, which it closes with the following paragraphs:

In New York and elsewhere a number of societies have long since abandoned a code of ethics as an unnecessary relic of the past, but we believe that to the Cleveland Medical Society belongs the honor of being the first society to vote to admit reputable practitioners of all stripes to membership. It is not a little significant that the first society to take this step should be one organized something over two years ago by young men, which has grown more rapidly, which had a larger average attendance at its meetings than any other society in the country and which is really the most flourishing medical society in the United States. While this long forward step may very likely be followed by reaction in some quarters, there is no reason to doubt that it is merely the first of a number of similar steps by other medical societies, or that finally the whole profession will follow in the lead of the Cleveland physicians.

We extract some of the details of the meeting, as published in the *Western Reserve Medical Journal*, by which it will be seen that Dr. X. C. Scott, a member of the judicial council of the American Medical Association, arrays himself on the side of progression:

The following resolution was then introduced by Dr. X. C. Scott:

WHEREAS, The constitution and by-laws of the Cleveland Medical Society provide that any legal medical practitioner is eligible to membership in said society; and

WHEREAS, At the last meeting the amendment which was proposed to make the constitution and by-laws of said Cleveland Medical Society to conform to the constitution, by-laws and code of ethics of the American Medical Association was rejected and laid on the table—

*Resolved*, That the active membership of said Cleveland Medical Society shall be open to any legal practitioner of this city, no distinction being made in regard to the school of medicine to which said legal practitioner may belong.

The adoption of the resolution was moved by Dr. P. Maxwell Foshay and seconded by Dr. Henry S. Upson. Dr. Foshay welcomed Dr. Scott to the ranks of the liberals and read an extract from the proceedings of the Mississippi Valley Medical Association, showing similar liberal stand taken by that body under Dr. Scott's presidency. The president, Dr. William E. Wirt, said the resolution was in conflict with the present constitution, which permitted only "regular" physicians to become members. He said the society must first place a construction upon the word "regular" before the resolution could be voted upon. Dr. R. M. Woodward said the United States surgeon-general had construed the word to mean "physician" in the usually accepted meaning of that term, without respect to creed or code. Dr. Upson moved that the society construe the word "regular" as any legally qualified practitioner of medicine, and the motion was adopted. Dr. O. T. Thomas thought that in view of this construction of the word "regular" the adoption of the resolution would be useless and moved that it be tabled. The original resolution was then put and carried with only one negative vote. The society then adjourned.

---

Following close upon the electric street car ambulance, recently put into commission by the St. Louis health department, comes the announcement that the Savannah, Florida & Western railway has adopted a new hospital car, recently described and illustrated in the *Columbus Medical Journal.* The car has a wide door at the side for the admission of patients, is provided with a properly equipped operating room and possesses ample accommodations for the reception and care of the injured. Commenting on this subject the *Chicago Medical Recorder* says that this road is the first to equip and put into service a hospital car. This ought to bring chagrin to our Northern railways, some of which ought to be leaders in everything that improves the transportation of sick or injured employes or travelers. To Dr. F. H. Caldwell, surgeon in chief of the railroad referred to, is due the credit, we believe, of taking the initiative in bringing about this reform in the humane, prompt and efficient transportation and care of sick and injured railway unfortunates.

---

We conclude in this number the report of the eighth periodical International Ophthalmological Congress, which has been running through several numbers of the Journal and was prepared by Dr. A. A. Hubbell, of Buffalo, from notes made at the Congress. This is, without doubt, the most complete report of any special medical congress held abroad, published in an American journal, and is attracting the attention of ophthalmologists throughout the country. It is being republished in the *Ophthalmic Record,* Nashville, Tenn.

---

The Journal takes pleasure in introducing to its readers two additional members of its editorial staff. Dr. Ernest Wende, clinical professor of dermatology in Buffalo University Medical College and health commissioner of Buffalo, is well known to the profession in this region. So, also, is Dr. A. A. Hubbell, professor of diseases of the eye and ear in Niagara University Medical College. These gentlemen have frequently contributed to our columns, and, we doubt not, this augmentation will increase the efficiency of the JOURNAL as well as be most acceptable to our subscribers.

## Figures and Tables

**Figure f1:**
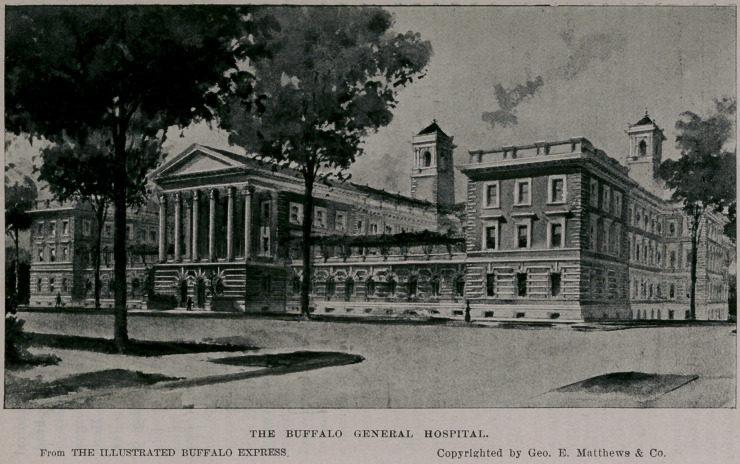


**Figure f2:**